# The Alberta Heart Failure Etiology and Analysis Research Team (HEART) study

**DOI:** 10.1186/1471-2261-14-91

**Published:** 2014-07-25

**Authors:** Justin A Ezekowitz, Harald Becher, Israel Belenkie, Alexander M Clark, Henry J Duff, Matthias G Friedrich, Mark J Haykowsky, Jonathan G Howlett, Zamaneh Kassiri, Padma Kaul, Daniel H Kim, Merril L Knudtson, Peter E Light, Gary D Lopaschuk, Finlay A McAlister, Michelle L Noga, Gavin Y Oudit, D Ian Paterson, Hude Quan, Richard Schulz, Richard B Thompson, Sarah G Weeks, Todd J Anderson, Jason RB Dyck

**Affiliations:** 1 Mazankowski Alberta Heart Institute, 2C2 WMC, 8440-112 Street, Edmonton, AB, Canada; 2 Libin Cardiovascular Institute of Alberta, Calgary, AB, Canada; 3 Department of Cardiac Sciences, University of Calgary, Calgary, AB, Canada; 4 Department of Medicine, University of Alberta, Edmonton, AB, Canada; 5 Department of Pediatrics, University of Alberta, Edmonton, AB, Canada; 6 Faculty of Nursing, University of Alberta, Edmonton, AB, Canada; 7 Department of Radiology, University of Calgary, Calgary, AB, Canada; 8 Departments of Medicine and Radiology, Montreal Heart Institute, Université de Montréal, Montréal, QC, Canada; 9 Faculty of Rehabilitation Medicine, University of Alberta, Edmonton, AB, Canada; 10 Department of Physiology, University of Alberta, Edmonton, AB, Canada; 11 Alberta Diabetes Institute, University of Alberta, Edmonton, AB, Canada; 12 Department of Pharmacology, University of Alberta, Edmonton, AB, Canada; 13 Department of Radiology and Diagnostic Imaging, University of Alberta, Edmonton, AB, Canada; 14 Department of Biomedical Engineering, University of Alberta, Edmonton, AB, Canada

**Keywords:** Heart failure, Preserved ejection fraction, Heart failure risk factors, Heart failure diagnosis

## Abstract

**Background:**

Nationally, symptomatic heart failure affects 1.5-2% of Canadians, incurs $3 billion in hospital costs annually and the global burden is expected to double in the next 1–2 decades. The current one-year mortality rate after diagnosis of heart failure remains high at >25%. Consequently, new therapeutic strategies need to be developed for this debilitating condition.

**Methods/Design:**

The objective of the Alberta HEART program (http://albertaheartresearch.ca) is to develop novel diagnostic, therapeutic and prognostic approaches to patients with heart failure with preserved ejection fraction. We hypothesize that novel imaging techniques and biomarkers will aid in describing heart failure with preserved ejection fraction. Furthermore, the development of new diagnostic criteria will allow us to: 1) better define risk factors associated with heart failure with preserved ejection fraction; 2) elucidate clinical, cellular and molecular mechanisms involved with the development and progression of heart failure with preserved ejection fraction; 3) design and test new therapeutic strategies for patients with heart failure with preserved ejection fraction. Additionally, Alberta HEART provides training and education for enhancing translational medicine, knowledge translation and clinical practice in heart failure. This is a prospective observational cohort study of patients with, or at risk for, heart failure. Patients will have sequential testing including quality of life and clinical outcomes over 12 months. After that time, study participants will be passively followed via linkage to external administrative databases. Clinical outcomes of interest include death, hospitalization, emergency department visits, physician resource use and/or heart transplant. Patients will be followed for a total of 5 years.

**Discussion:**

Alberta HEART has the primary objective to define new diagnostic criteria for patients with heart failure with preserved ejection fraction. New criteria will allow for targeted therapies, diagnostic tests and further understanding of the patients, both at-risk for and with heart failure.

**Trial registration:**

ClinicalTrials.gov NCT02052804.

## Background

Cardiovascular disease is one of the primary causes of death and disability in Canada and is a leading cause of death worldwide (http://www.who.int/chp/chronic_disease_report/en/). While advancements in medical and surgical therapies have improved cardiac mortality, these advances have led to an increase in the incidence and prevalence of heart failure (HF) [1]. At the national level, symptomatic HF affects 1.5-2% of Canadians, incurs $3 billion in hospital costs annually, and the global burden is expected to double in the next 1–2 decades [2,3]. In addition, the current one-year mortality rate after diagnosis of HF remains disturbingly high at >25% [4,5], and the condition has the worst effect on quality of life of any chronic medical condition [6]. Consequently, new therapeutic strategies need to be developed for this debilitating condition.

HF is a clinical syndrome characterized by specific symptoms (e.g. shortness of breath and reduced exercise capacity) as well as physical examination findings. HF has traditionally been described by a depressed ejection fractions ranging from <30-45% with <40% used as a criteria that is generally used in clinical trials and guidelines [7-9]. However, up to 50% of patients presenting with HF have preserved systolic function and are thus diagnosed with “diastolic HF” [3,10,11]. It is now recognized that a diverse group of cardiac diseases give rise to this condition and thus the clinical and research community has more appropriately renamed “diastolic HF” as HF with a preserved ejection fraction (HF-PEF) or HF-preserved systolic function (HF-PSF). Regardless of the nomenclature, no therapy has been proven in randomized clinical trials to reduce the morbidity or mortality of HF-PEF and, as such, no guideline endorses a specific therapy targeted at HF-PEF [8,9,12,13]. While the lack of an available agent is multi-factorial, we believe that the current criteria used for defining HF-PEF are inadequate. This may skew patient stratification in HF trials and prevent the identification of therapies that may actually have benefit in HF-PEF.

The objective of the Alberta HEART program (http://albertaheartresearch.ca) is to develop novel diagnostic, therapeutic and prognostic approaches to patients with HF-PEF. We hypothesize that novel imaging techniques and biomarkers will aid in describing HF-PEF. Furthermore, the development of new diagnostic criteria will allow us to: 1) better define the risk factors associated with the HF-PEF; 2) elucidate the clinical, cellular and molecular mechanisms involved with the development and progression of HF-PEF; 3) design and test new therapeutic strategies for patients with HF-PEF. Alberta HEART additionally provides training and education program for enhancing translational medicine, knowledge translation and clinical practice in heart failure.

## Methods/design

### Trial status

The study is approved by the Health Research Ethics Boards at the University of Alberta, Covenant Health and the University of Calgary. Recruitment began in January 2010 and as of March 31, 2014, 649 patients have been enrolled. ClinicalTrials.gov NCT02052804.

### Study design

This is a prospective observational cohort study of patients with, or at risk for, HF.

### Study population

Patients are adults (i.e. aged over 18 years of age) recruited from Alberta (population 4.0 million), principally in Edmonton and Calgary but also from rural regions. In order to enroll and study patients across the spectrum of risk of developing HF, with signs or symptoms mimicking HF, or with a diagnosis of HF, a broad recruitment strategy that categorizes patients into 5 distinct groups is underway. The 5 distinct groups are:

1. High-risk of developing HF-PEF but no clinically overt HF: Patients will have one or more of the following: hypertension (≥3 medications or LVH on ECG or left ventricular mass index > gender-matched upper limit normal on an imaging test); diabetes (>45 years of age); atrial fibrillation; or obesity (body mass index >30). Patients will be generally asymptomatic (no dyspnea or fatigue) and have no known prior HF or other cardiovascular disease.

2. High-risk of developing HF-PEF with other clinically overt disease: Patients with other cardiovascular diseases will be recruited including atrial fibrillation, chronic coronary artery disease (including those with a recent acute coronary syndrome > 2 weeks prior), and COPD.

3. Patients with known HF-PEF: These are patients who have been diagnosed with HF-PEF based on the clinical phenotype of symptoms consistent with HF, and a preserved ejection fraction. Patients with the clinical diagnosis of HF-PEF and an ejection fraction >45% will be enrolled in this study from the heart failure clinics in Alberta. For further definition, the group will be divided into 3a (LVEF always ≥ 45%; no right ventricle involvement), 3b (LVEF previously <45%, now ≥ 45%; with or without right ventricle involvement), and 3c (LVEF always ≥ 45%; right ventricle involvement or pulmonary hypertension (TAPSE <16 mm, PASP > 35 mmHg, or clinical judgment)).

4. Patients with known systolic HF: Patients with systolic HF often have abnormalities in the mechanical diastolic function and are necessary to complete the spectrum to act as a comparator for group 3. An ejection fraction <45% and a diagnosis of HF will be included.

5. Healthy age- and gender-matched controls: Patients with no evidence of coronary artery disease, hypertension, diabetes mellitus, organ disease or replacement therapies; no evidence of inflammatory or autoimmune conditions and not on cardiac medications.

**Figure 1:**
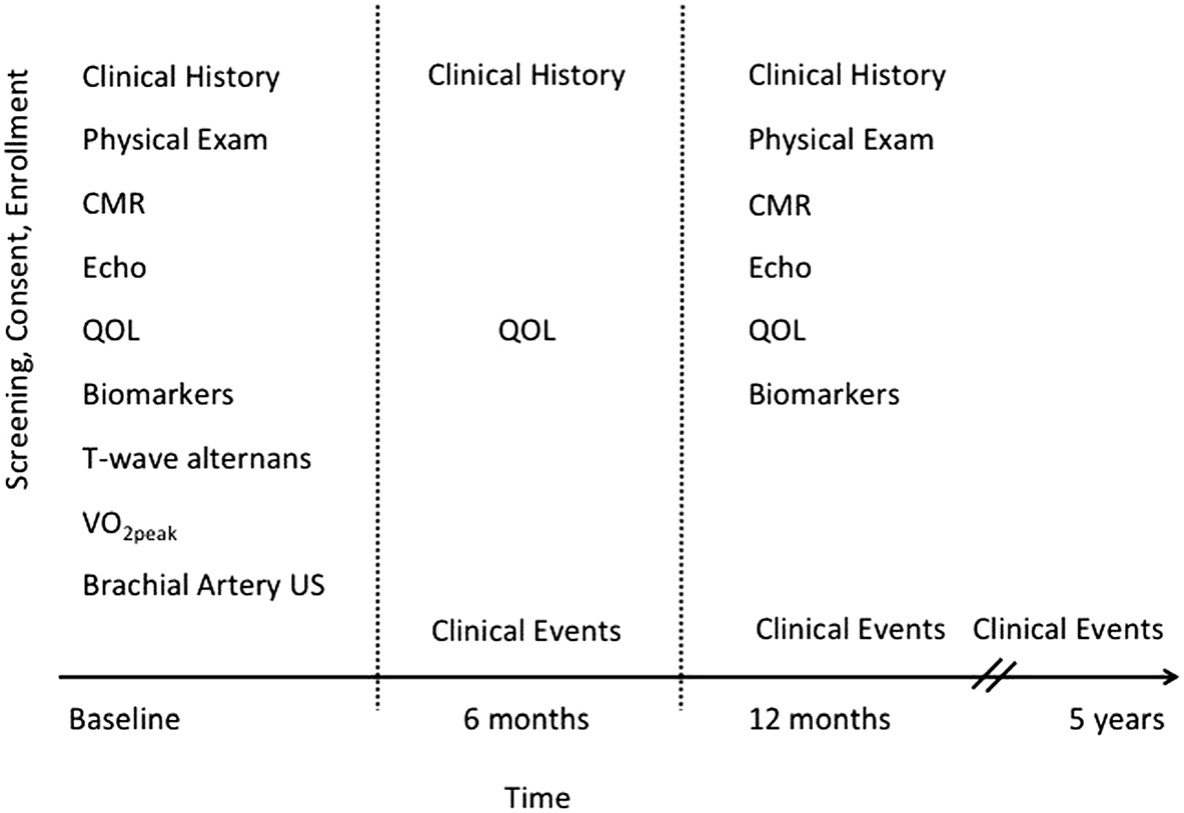
Alberta HEART study design.

Patients will be recruited from HF and other clinics across Alberta. Control patients are recruited through referrals from patients, clinicians and the broader community across Alberta via public advertising, media events and other public engagement. All patients signed informed consent and the study is approved by the Health Research Ethics Boards at the University of Alberta, Covenant Health and the University of Calgary. After consent, patients are enrolled and undergo comprehensive clinical, quality of life, biomarker, electrophysiologic and imaging assessments (Figure 1). Patients are also eligible for substudy enrollment (see below). Patients are re-assessed at 6 and 12 months and longer-term clinical and outcome surveillance is planned. Data is managed on the Alberta Provincial Project for Outcome Assessment in Coronary Heart Disease platform (http://www.approach.org/).

### Adjudication

Given the lack of a single accepted and validated definition for HF-PEF, the 3 most commonly used clinical criteria from landmark papers [14-16] ongoing or completed trials [17,18] or population studies [3,10,11] will be evaluated for applicability to the development of new criteria (Table 1). These criteria highlight two important points: (1) diagnostic uncertainty may exist and should be captured by any criteria proposed using a hierarchical methodology; and (2) time of evaluation may alter the certainty of the diagnosis as clinical findings (and non-invasive or invasive markers) may change with therapy or time. In addition, none of these 3 sets of criteria have been compared, or evaluated in the setting of other conditions, which may mimic symptoms of HF (e.g. lung disease, hypertension, diabetes, obesity, deconditioning).

**Table 1:** Current diagnostic criteria for HF-PEF

**Definition**	**ESC **[12,14]	**Vasan et al. **[15]	**Zile et al.**[16,19]
**Signs or symptoms?**	Symptoms and signs of HF	Symptoms and signs, supporting laboratory tests and a typical clinical response to treatment^#^	Symptoms and signs of HF (by Framingham criteria)
**Ejection fraction**	LVEF > 50% and LVEDVI < 97 mls/m^2^	LVEF ≥ 50% within 72 hours of HF event**	LVEF > 50%
**Echo/Cath/Biomarker evidence of diastolic dysfunction?**	Yes	Yes***	Not required
		Abnormal LV relaxation, filling, distensibility indices on cardiac catheterization	

^#^with diuretics, with or without documentation of elevated LV filling pressure (at rest, on exercise, or in response to a volume load) or a low cardiac index; **downgraded to “Probable” if EF not defined in first 72 hours; ***downgraded to “Possible” if no Echo or Cath evidence; ESC, European Society of Cardiology.

**Figure 2:**
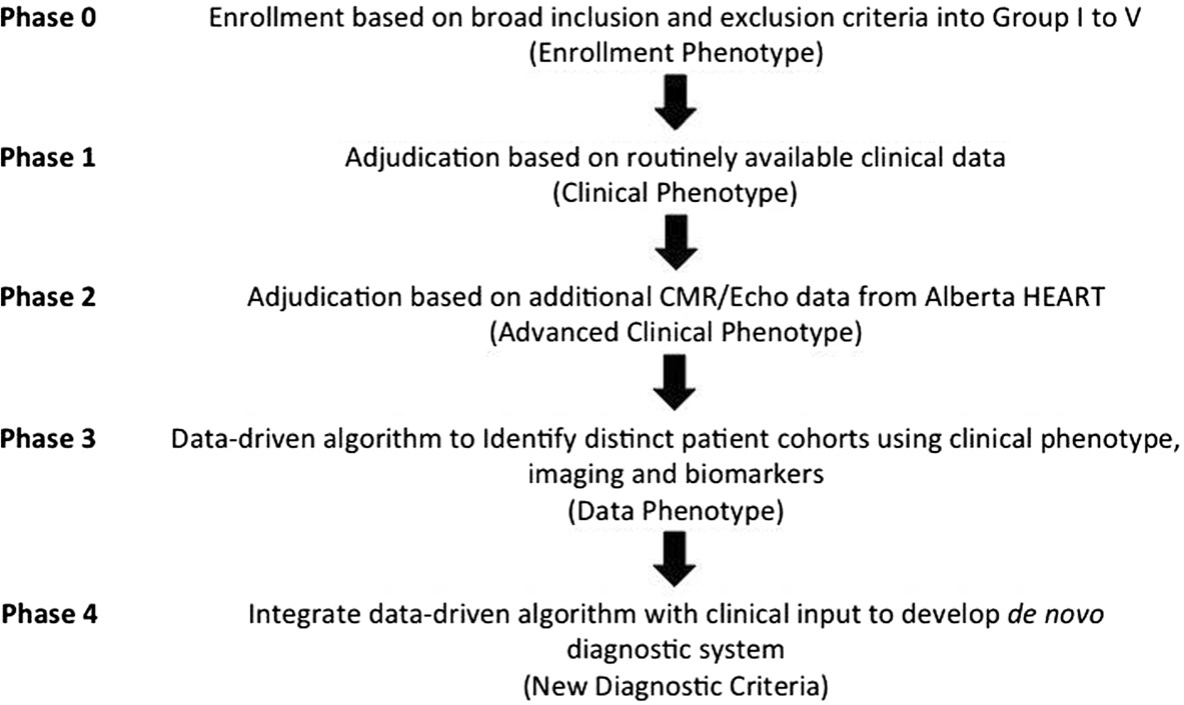
Alberta HEART adjudication process.

Each of the three definitions will be applied to the cohort in order to evaluate the sensitivity, specificity, diagnostic accuracy, positive predictive value and negative predictive value using the diagnostic testing model based on Bossuyt *et al.*[20] and Sackett and Haynes [21] and the reporting methods endorsed by STARD [22] and QUADAS-2 [23]. A reference standard for HF-PEF is currently absent. Therefore, we have chosen to use a consensus panel approach using a panel of adjudicators blinded to the group of original recruitment (Figure 2).

#### Phase 1

In the first phase, the adjudicators will be asked to group the participants into one of the 5 groups above, using pre-enrollment (i.e. no study related variables) clinical criteria. We will then test the current definitions as to their validity.

#### Phase 2

In the second phase, information from the study echocardiogram and cardiac MRI will be used to re-adjudicate participants and explore if the clinical phenotype can be better enhanced using standardized imaging biomarker information.

#### Phase 3

In the third phase, data from the clinical phenotype, novel imaging markers, and biomarker testing to see if it can identify distinct patient cohorts. This will also incorporate novel imaging or biomarkers that are emerging or have not been previously validated.

#### Phase 4

In the final phase, all of the above will be integrated to develop a *de novo* diagnostic system via a panel-based method. Potential criteria will be viewed singly and together in order to develop a novel diagnostic scoring system with the following principles: (1) clinical symptoms and signs must be included as a basis for the diagnosis of HF-PEF; (2) diagnostic certainty should be categorized into definite, probable, possible, and not present; (3) time of evaluation must be included with respect to acute events; (4) no binary LVEF cutoff exists therefore a spectrum of LVEF will be used; (5) areas to be considered as candidate variables for evaluation should include mechanical, electrical, biomarker, and imaging modalities and should be done at rest and with provocation of exercise; (6) subtypes of HF-PEF likely exist based on etiology or mechanism of dysfunction and these subtypes likely include, but are not limited to, ventricular-vascular decoupling, myocyte dysfunction, infiltration, valve-related, extracellular matrix hypertrophy**,** post-operative and arrhythmia-related, especially atrial fibrillation; and (7) a simple clinical tool will be adopted if valid and clinically relevant, so attention to the end-user will be incorporated at an early stage through interaction with end-users (see http://www.albertaheartresearch.ca). External validation will be sought in existing datasets including MAGGIC, CHARM and the Olmsted County database.

### Imaging– MRI and echocardiography

Most diagnostic constructs for the identification of HF-PEF, such as those from the European Society of Cardiology (Table 1) [14], employ imaging-measures of diastolic function in a diagnostic algorithm. However, these are used as binary variables and cut-points considered important for the diagnosis of HF-PEF. Several new quantitative imaging tests are available that more directly measure the physical mechanisms during ventricular relaxation that are associated with diastolic function, such as ventricular untwisting and global tissue strain rates, or that reflect tissue characteristics including diffuse and focal fibrosis and edema.

Thus, our goal is to evaluate a wider array of imaging tests for the purpose of defining a new set of practical criteria for the characterization of diastolic dysfunction that would be widely available. In addition to standard measures, main features will be evaluated using one or both of echocardiographic and CMR imaging: (1) ventricular stiffness [24,25]; (2) ventricular relaxation [24,26]; and (3) ventricular-vascular coupling [27,28]. This new approach will address the significant variability (often due to load dependence) and low specificity of the conventional imaging parameters of diastolic function and develop load-independent measures.

In brief, the CMR scans will be performed on a 1.5 T magnet (Sonata, Siemens and Avanto, Siemens) and will include an assessment of: (1) atrial and ventricular volumes and function using steady state free precession (SSFP), (2) myocardial tissue characterization using late gadolinium enhancement (LGE) as well as quantitative T1 and T2 imaging, (3) estimation of ventricular and vascular stiffness using SSFP, phase contrast and tagging techniques and (4) pulmonary water content. For LGE and T1 imaging, 0.15 mmol/kg of gadolinium contrast (Gadovist, Bayer) will be given intravenously. Cardiac volumes and function and myocardial scar will be analyzed using commercially available software (Syngo, Siemens and CMR42, Circle) by experienced interpreters blinded to the study group assignment. Other measures of cardiac function and myocardial tissue characterization as well as vascular function and pulmonary water content will be performed using custom built software (MATLAB).

2D and 3D echocardiography will be performed on IE 33 (Philips) scanners with and without intravenous injection of the ultrasound contrast agent Definity. In addition, recordings using color, spectral, tissue Doppler and speckle tracking imaging will be obtained according to the American Society of Echocardiography (ASE). The following measurements will be performed by two experienced observers according ASE standards: LV volumes, ejection fraction, LA volumes, RV size and function, quantification of valvular lesions, systolic pulmonary artery pressure, mitral/tricuspid inflow velocities, mitral/tricuspid annular velocities, global longitudinal strain and strain rate. The recordings will also be processed using custom built software for fusion of 3D datasets and new tools to assess myocardial deformation which promise a more reliable assessment of volume changes as well as systolic and diastolic LV/RV function.

### Exercise physiology substudy

A cardinal feature of HF, even when well compensated, is decreased exercise capacity (VO_2peak_) that is due, in part, to impaired cardiovascular and skeletal muscle that results in reduced convective or diffusive O_2_ transport or decreased O_2_ utilization by the active muscles [29,30]. Currently, no study has prospectively examined the determinants of exercise intolerance across the HF continuum (i.e. healthy individuals and AHA/ACC stages A to C), therefore, uncertainty remains regarding the role that abnormal cardiac, vascular or skeletal muscle function play in limiting peak VO_2_ across the health, at risk and HF continuum.

The goal of the substudy is to compare peak VO_2_ (and its determinants) in healthy individuals, individuals at risk for HF and clinically stable individuals with HF-REF and HF-PEF. Subjects will be drawn from Groups 1 to 5 (above) and will undergo a cardiopulmonary exercise test on a Lode (Groningen, NL) cycle ergometer (resting, with 15 W increase in peak power output every two minutes until volitional exhaustion) with expired gas analysis and inert gas rebreathing (Innocor CPX, Odense, DK) [31] to measure peak VO_2_ and its Fick determinants (i.e. peak heart rate, stroke volume, cardiac output, systemic vascular resistance, arterial-venous oxygen difference). We will also use a non-invasive multisensory accelerometer (SenseWear Armband) to measure Daily Energy Expenditure (DEE), which will provide information on how physical activity measurements relate to established prognostic parameters. Assessing physical activity in patients doing their usual activities allows for day-to-day fluctuations to be taken into account. This will objectively assess daily physical activity, sedentary time and energy expenditure and compare these measures to established HF prognostic parameters.

In addition, the role that vascular function (MRI-derived aortic distensibility, brachial artery flow mediated dilation) and ventricular-arterial coupling abnormalities may play in limiting exercise performance will also be determined.

### Vascular substudy

The endothelium plays a key role in vascular homeostasis through the release of a variety of autocrine and paracrine substances [32,33]. Dysfunction of endothelial cells is a systemic process and the initiating event in atherosclerosis. This has adverse effects on afterload and ventricular-vascular mismatch may occur. Over the past two decades it has been clearly demonstrated that subjects with systolic dysfunction have impairment of endothelial function. It was recently demonstrated that subjects with HF-PEF did not have attenuated conduit vessel endothelial dysfunction compared with age-matched controls [34]. Exercise training in this group improved VO_2max_ but not large vessel endothelial function [35]. However, another group demonstrated attenuation of microvascular function in a cohort with HF-PEF and was able to demonstrate an association of microvascular function with outcomes [36]. We will recruit: (a) control subjects with no signs or symptoms of HF; (b) subjects with HF and systolic dysfunction due to dilated cardiomyopathy (EF <35% and no coronary artery disease) and no active vascular risk factors; and (c) subjects with HF-PEF in Group 3 (above) and no active vascular risk factors. Subjects will undergo the following non-invasive assessments of vascular health: a) brachial flow-mediated dilation, b) hyperaemic velocity, and c) finger-tip pulse arterial tonometry (PAT). These measure conduit vessel endothelial function (FMD), microvascular function (hyperaemic velocity), fingertip small vessel endothelial function and capacitance (PAT). Details of the methodology are well published and utilized by our group [37,38].

### Blood pressure wave substudy

Cardiac catheterization is indicated for clinical reasons in a subset of patients enrolled into Alberta HEART. These patients will undergo an additional set of high fidelity simultaneous pressure and flow measurements using a combined Doppler/Millar pressure wire (Combiwire®- Seimens) at 5 pre-specified locations in the aorta (vena contracta, mid-aortic arch, distal to left subclavian, at the diaphragm and infrarenal). Using this data, we will validate the Reservoir-Pressure theory of pressure/flow propagation in humans (already validated in large animal models). The successful validation of this approach will enable us to predict, using a derived mathematical formula, the effects of antihypertensive agents (both existing and new) as well as future device interventions upon individual pressure dynamics. We also hope to determine characteristics of the pressure/volume wave to potentially predict the development of future vascular de-coupling and/or ventricular remodeling.

### Biomarkers substudy

The use of biomarkers will allow us to screen high-risk populations, to create a risk-prediction model in patients with HF-PEF and to provide new insights into the pathophysiology of HF-PEF. We hope to distinguish between HF-PEF and HF-REF and to provide prognostic insight into those with overt disease with the ultimate aim of providing tailored therapy to these different patient populations [39,40]. Importantly, we will be able to link these data to important clinical outcomes such as mortality, survival free of heart transplantation, and hospitalizations. These biomarkers will also be linked with our findings from vascular physiology and imaging assessment. Serum, plasma samples and whole blood will be collected and stored for analysis in our core biomarker facilities. Biomarker testing will encompass both hypothesis-generating and hypothesis-testing approaches. An “omics” platform will be used, including microarray (mRNA and microRNA), proteomics and metabolomics with a systems-based analysis. A more tailored biomarker analysis of the extracellular matrix, natriuretic peptides, inflammatory biomarkers and other novel biomarkers will also be undertaken. We will collaborate with the Prevention of Organ Failure Centre of Excellence (PROOF; http://www.proofcentre.ca) at the University of British Columbia, which is cross-disciplinary team that unites scientific, technical, and commercialization expertise in order to implement biomarkers to improve management of heart, lung and kidney disease.

### Risk substudy

Patients will undergo non-invasive risk evaluation using locally developed technology [41]. The technology is the antithesis of a Holter monitor, as instead of randomly acquiring data, signals are obtained during scripted interventions designed to elicit physiologic transients, which unmask physiologic defects in auto-regulation. Using this data acquisition system, 2 metrics of risk of death were discovered and partially validated. In contrast to the current study, the original pilot study only recorded a single bipolar ECG whereas we now record 6 standard ECG leads. This will provide a better opportunity to examine spatial heterogeneity of T wave alternans.

### Clinical outcomes

Patients will have sequential testing including quality of life and clinical outcomes over the first 12 months of study entry. After that time, we will passively follow study participants via linkage to external administrative databases held by Alberta Health. Clinical outcomes of interest include death, hospitalization, emergency department visits, physician resource use, and/or heart transplant. Patients will be followed for a total of 5 years of follow-up.

Recognizing that our prospectively-enrolled cohort will be subject to selection and referral biases, we also plan to use administrative data maintained by Alberta Health and Alberta Health Services to explore health service use and outcomes in all Albertans with HF. This will be used in two ways:

1. Patients who have provided consent in the prospectively collected cohort will be tracked longitudinally to capture all of their interactions with the healthcare system.

2. Anonymized data obtained from Alberta Health will be used to explore the in-patient, out-patient, emergency department, medications and outcomes of all patients with HF using techniques previously validated by our group [1,5,42,43].

These complementary approaches will allow for appropriate comparisons of patient-, provider- and system-level variables that may vary across the spectrum of HF or those patients at risk for HF.

### Sample size

Alberta HEART plans to recruit 700 patients with up to 100 patients in each of Groups 1, 2 and 5 and 200 patients in each of Groups 3 (HF-PEF) and 4 (known systolic HF). Given the lack of validated data from which to calculate a sample size, a convenience sampling technique has been used, recognizing that for some markers (e.g. imaging, exercise, biomarkers) the HEART study will be significantly powered, but for other outcomes (e.g. clinical outcomes) HEART will be underpowered. Nevertheless, the sample size will be increased as appropriate to meet the primary objectives of the study. An additional 100 age- and gender-matched controls (matched to group 3) will be been recruited and tested. Recruitment began in January 2010 and as of March 31st, 2014, 649 participants have been enrolled.

### Training program

Alberta HEART has a training program (Supporting Training Encompassing All in Diastolic Heart Failure [STEADi HF]) to train researchers of all backgrounds and across all 4 pillars of research as described by the Canadian Institutes of Health Research (http://www.cihr.ca). These competitive training grants are awarded to trainees who are researching an area of heart failure and are collaborating with/mentored by another team member. This will assist in the building of interdisciplinary partnerships along with providing the trainee with a clearer understanding of the research skills in another discipline.

### Discovery science

Of a total of 24 investigators, Alberta HEART includes 8 scientists who have a primary focus on traditional fundamental discovery science. The project also incorporates aspects important for translational science: development of an appropriate animal model from which to test novel therapeutics before first-in-man studies; studying the tissue, cellular and sub-cellular interactions that may be related to a clinical phenotype; and exploring proteomic, metabolomic and genomic pathways to understand overall cardiac function.

## Discussion

Alberta HEART has the primary objective to define new diagnostic criteria for patients with HF-PEF. New criteria will allow for targeted therapies, diagnostic tests and further understanding of the patients, both at-risk for and with heart failure. Interaction across pillars of research and with clinicians, patients, the public and policy makers is embedded within this project to ensure accurate and timely translation of new and existing knowledge for the prevention, diagnosis and treatment of patients with cardiovascular disease.

## Abbreviations

ACC: American College of Cardiology; AHA: American Heart Association; ASE: American Society of Echocardiography; CMR: Cardiac magnetic resonance; COPD: Chronic obstructive pulmonary disease; DEE: Daily energy expenditure; ECG: Electrocardiogram; EF: Ejection fraction; FMD: Flow-mediated dilation; HEART: Heart Failure Etiology and Analysis Research Team; HF: Heart failure; HF-PEF: Heart failure with preserved ejection fraction; HF-PSF: Heart failure with preserved systolic function; LA: Left atrium; LGE: Late gadolinium enhancement; LV: Left ventricle; LVEF: Left ventricular ejection fraction; MRI: Magnetic resonance imaging; PASP: Pulmonary artery systolic pressure; PAT: Pulse arterial tonometry; QUADAS-2: Quality assessment of diagnostic accuracy studies 2; RV: Right ventricle; SSFP: Steady state free precession; STARD: STAndards for the reporting of diagnostic accuracy studies; STEADi HF: Supporting training encompassing all in diastolic heart failure; TAPSE: Tricuspid annular plane systolic excursion.

## Competing interests

Funding for Alberta HEART is provided by Alberta Innovates – Health Solutions (Grant # AHFMR ITG 200801018) awarded in 2009. Additional funding is provided by NCE CECR Centre of Excellence for Prevention of Organ Failure and the Alberta Diabetes Institute. In-kind contributions were received from Capital Health Regional Authority (now Alberta Health Services) and the Alberta HEART investigators. JAE, JRBD, FAM, HJD, GDL, GYO and TJA receive salary awards from AI-HS. GYO receives salary awards from HSFC and CIHR. PEL holds the Charles A. Allard Chair in Diabetes Research. FAM holds the University of Alberta Chair in Cardiovascular Outcomes Research. MF holds the Hornstein Chair in Cardiovascular Imaging. HB holds the Heart & Stroke Foundation Endowed Chair for Cardiovascular Research. TA holds the Merck Frosst Chair in Cardiovascular Research, University of Calgary.

## Authors’ contributions

All authors were involved in the conception and design of the research program. TJA, HB, AMC, HJD, JRBD, JAE, MJH, JGH, FAM, GYO, DIP and RBT contributed to the writing of the manuscript. All authors read and approved the final manuscript.

## Pre-publication history

The pre-publication history for this paper can be accessed here:

http://www.biomedcentral.com/1471-2261/14/91/prepub
